# 393. Healthcare and Out-of-Pocket Costs Associated With *Clostridioides difficile* Infection Among US Adults 18–64 Years of Age

**DOI:** 10.1093/ofid/ofac492.471

**Published:** 2022-12-15

**Authors:** Holly Yu, Tamuno Alfred, Jingying Zhou, Jennifer Judy, Margaret A A Olsen

**Affiliations:** Pfizer Inc., Collegeville, Pennsylvania; Pfizer Inc, New York, New York; Pfizer Inc, New York, New York; Pfizer, New York, New York; Washington University School of Medicine, St. Louis, Missouri

## Abstract

**Background:**

Limited data are available on healthcare costs associated with *Clostridioides difficile* infection (CDI) among adults < 65 years of age.

**Methods:**

This retrospective cohort study used Optum’s de-identified Clinformatics® Data Mart to identify first CDI episodes from 2016–2019 among individuals 18–64 years of age insured under commercial plans. CDI was defined by ICD9/ICD10 diagnosis codes or a combination of CDI diagnosis/testing with antibiotic treatment. Healthcare costs were evaluated among CDI+ cases and 1:1 propensity score-matched CDI− controls. Both CDI+ cases and controls had continuous database enrollment for ≥12 months prior; follow-up continued through the earliest of death, disenrollment or 12 months post-index. Costs were analyzed by age group, acquisition type, and hospitalization status within 2 months of the CDI index date.

**Results:**

We identified 13,820 CDI+ cases and 4,027,386 potential controls; 12,999 cases (18–49 y, n=6667; 50–64 y, n=6332) were matched to a control. In the 50–64-year matched groups, mean total healthcare costs at 2 months post-index were $18,453 (CDI+) and $6819 (CDI−); $11,634 in excess costs were attributable to CDI (**Figure 1A**). Mean attributable costs were higher for hospitalized healthcare-associated ($68,745) and hospitalized community-associated ($37,646) cases than non-hospitalized healthcare- or community-associated cases ($8333 and $2953, respectively). The mean attributable out-of-pocket (OOP) cost in the 50–64-year age group was $573 and was higher for community-associated vs healthcare-associated cases (**Figure 1B**). Overall CDI-attributable costs in the 18–49-year age group trended similarly but were mostly lower for total healthcare costs ($7826 [CDI+, $12,019; CDI−, $4193]; **Figure 2A**) and slightly higher for OOP healthcare costs ($642 [CDI+, $954; CDI–, $311]; **Figure 2B**).

**Figure:**
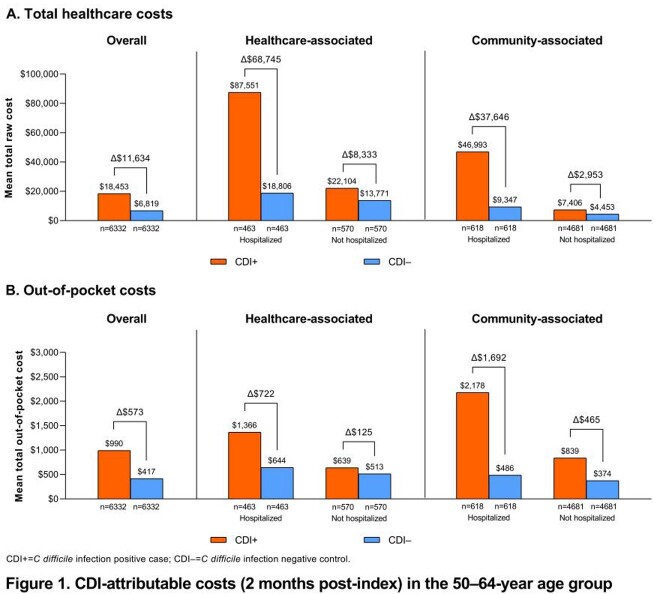

**Figure:**
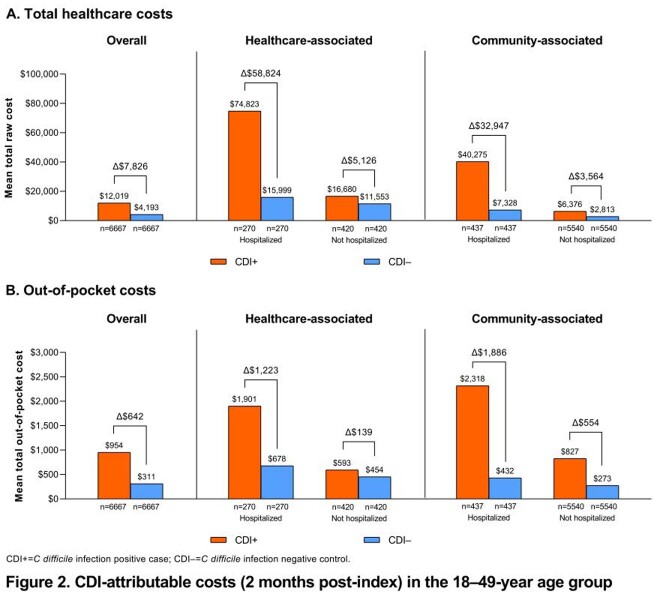

**Conclusion:**

CDI-attributable healthcare costs among US individuals 18–64 years of age are substantial, particularly for those hospitalized for CDI. Effective prevention in younger adults may significantly reduce healthcare resource utilization and costs to both the healthcare system and patients.

Funded by Pfizer Inc.

**Disclosures:**

**Holly Yu, MSPH**, Pfizer Inc: Employee|Pfizer Inc: Stocks/Bonds **Tamuno Alfred, PhD**, Pfizer Inc: Employee|Pfizer Inc: Stocks/Bonds **Jingying Zhou, MA**, Pfizer Inc: Employee|Pfizer Inc: Stocks/Bonds **Jennifer Judy, MS, PhD**, Pfizer Inc: Employee|Pfizer Inc: Stocks/Bonds **Margaret A A. Olsen, PhD, MPH**, Pfizer Inc: Advisor/Consultant|Pfizer Inc: Grant/Research Support.

